# Computational methodology and molecular dynamics analysis of andrographolide bioactivity against *Cutibacterium acnes*

**DOI:** 10.3389/fchem.2025.1627758

**Published:** 2025-06-11

**Authors:** Min Lin, Lihui He, Guodong Ye, Xiaotian Zhao

**Affiliations:** ^1^ Department of Dermatology, Chengdu Second Peoples Hospital, Chengdu, China; ^2^ Department of Pharmacy, Chengdu Second Peoples Hospital, Chengdu, China; ^3^ National Medical Products Administration and State Key Laboratory, The Fifth Affi1iated Hospital, School of Pharmaceutical Sciences, Guangzhou Medical University, Guangzhou, Guangdong, China

**Keywords:** *Cutibacterium acnes*, andrographolide, density functional theory, molecular docking, molecular dynamics

## Abstract

*Cutibacterium acnes* (formerly known as *Propionibacterium acnes*) is a Gram-positive anaerobic bacterium that is frequently found in sebaceous glands and human skin hair follicles. Although it is a normal component of the skin microbiota, acne may result from its overgrowth in specific situations. Andrographis paniculate, a traditional Chinese herb, contains a diterpenoid compound called andrographolide (AGP), which has anti-inflammatory, antibacterial and antioxidant. AGP can strengthen the skin barrier functions by preventing the release of inflammatory factors like TNF-α and IL-6. It has also shown that its antibacterial qualities can inhibit pathogens such as *Cutibacterium acnes.* In this study, through the use of the quantum chemistry approach, we investigated AGP in DMSO and gas phase, in connection to the geometric foundations and energy dynamics by Density Functional Theory (DFT). Furthermore, this work has been carried out on the structural analysis by orbital examination and electrostatic potential (ESP) assessment. Subsequently, we derived the molecular interactions utilizing the independent gradient model within Hirshfeld molecular density partitioning (IGMH). Additionally, the stability and interaction of AGP and fusidic acid with the protein _CA_lipase of *Cutibacterium acnes* have been investigated by molecular docking and molecular dynamics simulations. This study might provide a fresh perspective for investigating an effective extraction process via methodological analytical discussions.

## 1 Introduction

*Cutibacterium acnes* (*C. acnes*), also named *Propionibacterium acnes,* is a gram-positive, anaerobic bacterium that resides in sebaceous-rich areas of the skin, such as the face, chest and back (Dréno et al., 2018). *C. acnes* plays a vital role in maintaining the health of the skin against preventing colonization by pathogenic organisms and breaking down skin oils to release free fatty acids (Yang et al., 2020). However, under dysregulated skin conditions such as increased sebum production and follicular occlusion, it can shift toward a pathogenic state and contribute significantly to acne vulgaris. Acne vulgaris is characterized by excess sebum production, hyperkeratinization, and follicular blockage, creating an anaerobic environment conducive to *C. acnes* proliferation (Svensson et al., 2018).

As *C. acnes* proliferates, it can release enzymes like lipases, hyaluronidase and neuraminidase which are essential virulence factors to hydrolyze sebum triglycerides into free fatty acids and glycerol, creating a nutrient-rich and immunologically active environment Tomida et al., 2013. The generated fatty acids not only support bacterial growth but also stimulate local inflammation by activating innate immune responses. In this process, skin acidity is maintained and this functions as a natural barrier from harmful pathogens, providing innate skin immunity (Kim et al., 2020). Structural studies reveal that these lipases (_CA_lipase) possess a hydrophobic lid covering the catalytic triad, which plays a key role in substrate binding and enzyme regulation (Kim et al., 2020) Furthermore, *C. acnes* has ability to form biofilm which can act as protective barriers that increase antibiotic resistance and immune evasion, making acne treatment more difficult (Coenye et al., 2007; Feuillolay et al., 2016).

Traditional therapies targeting *C. acnes*-related conditions typically involves topical and systemic therapies aimed at reducing bacterial load, minimizing inflammation and follicular blockage. Clindamycin and erythromycin were among the earliest topical antibiotics used. However, widespread use has led to increasing antibiotic resistance (Eady et al., 1989; Dessinioti and Katsambas, 2017; Sharma et al., 2003; Kim et al., 2017). Benzoyl peroxide (BPO) works by releasing oxygen into the pores, creating an anaerobic environment. Due to its oxygen-releasing, anti-inflammatory properties and without inducing resistance, BPO has emerged as a preferred alternative for *C. acnes* (Dréno, B. 2016; Bowe and Kober, 2014; Sagransky et al., 2009). In addition, photodynamic therapy (PDT) and light-based treatments utilize bacterial porphyrin photosensitization to induce *C. acnes* death are explored as the therapeutic option (Wan and Lin, 2014; Barroso et al., 2021). Nevertheless, there remains a strong demand for novel, non-antibiotic therapeutic strategies with defined molecular mechanisms to treat *C. acne*.

Andrographolide is a biologically active diterpene lactone extracted from *Andrographis paniculate*. It is widely known for its anti-inflammatory, antioxidant, antibacterial, antiviral and anticancer effects (Okhuarobo, et al., 2014; Zhang et al., 2020; Mishra et al., 2007; Shen et al., 2002; Gupta et al., 2017). In recent years, AGP has attracted widespread attention from the scientific community due to its therapeutic potential in modern medicine. AGP has been shown to prevent biofilm formation, which may be effective against a variety of bacteria and pathogens, making AGP have significant antibacterial properties (Zhang et al., 2021). Furthermore, AGP inhibits the NF-κB signaling pathway, a key inflammation regulator, making it a potential candidate for treating acne and other inflammatory diseases (Berk et al., 2022; Hu et al., 2025; Banerjee et al., 2017).

**FIGURE 1:**
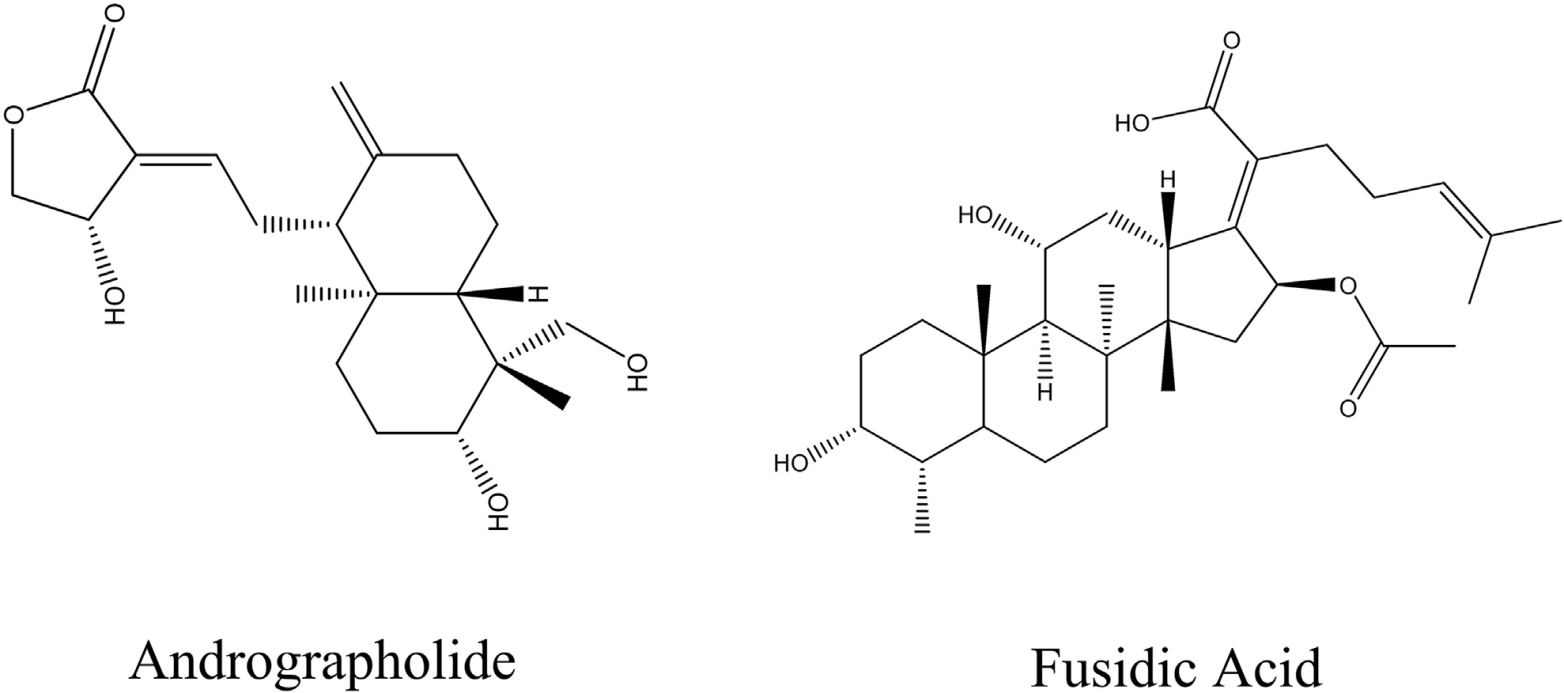
The depiction illustrates the chemical structures of AGP and FA.

In this present work, AGP and the fusidic acid (FA) (in Figure 1) were chosen as title compounds to evaluate the interaction with lipase of *C. acnes* (_CA_lipase). Analysis of both allyl sucrose ether and ordinary organic solvents can be achieved through the use of quantum chemical computations approaches, we present a brief overview of conceptual DFT analysis (Geerlings and Proft, 2008; Fayet et al., 2009; Chakraborty et al., 2013). The Gaussian 16 program and Gauss view 6.0 software were used in order to construct the most optimal geometrical structure of the chemical that was provided (Gaussian 16, 2016). The DFT approach was used to analyze the compound’s electrical characteristics via the Conceptual Density Functional Theory (Raghavachari, 2000; Kryachko and Ludeña, 2014). The electrostatic potential (ESP) and the electric surface area distribution studies from Multiwfn 3.8, a wavefunction analyzer (Lu and Chen, 2012a). To further explore noncovalent interactions, the Independent Gradient Model based on Hirshfeld partitioning (IGMH) was utilized (Lu and Chen, 2022), revealing specific van der Waals and hydrogen-bonding interactions between AGP and CAlipase. The analysis suggests that AGP has a strong binding affinity toward the active site of the enzyme, potentially obstructing substrate access and attenuating lipase activity. Dimethyl sulfoxide (DMSO) was used as a solvent due to its laboratory and industrial applications as solvents for many gases, synthetic fibres and natural products (Nasidi et al., 2024). Our findings highlight the potential of AGP as a non-antibiotic lipase inhibitor targeting C. acnes, providing new mechanistic insights into its antibacterial and anti-inflammatory action. This approach represents a novel application of traditional herbal medicine in dermatology, emphasizing structure-based drug design through quantum chemical descriptors and interaction modeling. By identifying molecular interactions at the electronic level, this work offers a new perspective for targeting virulence factors such as lipases in C. acnes, paving the way for future development of selective and resistance-free acne therapeutics.

## 2 Computational methods

The molecular structure computations were conducted using the Gaussian 16 program. The theoretical approach employed the M062X/def2TZVP method for geometry optimization of AGP and FA, with the IGMH portion utilizing the 6-311++g (d,p) basis set and the B3LYP functional (Świderski, et al., 2013). SMD (Solvent Model Density) (Dalessandro and Pliego Jr, 2018) was applied as the solvent model. Graphs were generated using Gaussian View 6 and Origin 8 software. The energy gap of the title compounds was determined by analyzing Frontier Molecular Orbitals (FMO). Wavefunction analysis was performed using the Multiwfn 3.8 program, which provided the wavefunction files and evaluated the electrostatic potential and electric distribution. Isosurface maps were created using VMD 1.9.3 (Humphrey et al., 1996) in conjunction with the output files from Multiwfn. Quantitative studies of the electrostatic potential on the van der Waals (vdW) surface were carried out in Multiwfn with a grid spacing of 0.15 bohr. The IGMH method was particularly useful for gaining insights into intermolecular interactions between specific amino acids and the title compounds.

Molecular docking was employed to predict the combined mode of AGP and FA with the protein 6KHW. As previously reported (Li, et al., 2021), the structure of 6KHW was retrieved from the Protein Data Bank (https://www.rcsb.org/). Subsequently, AGP and FA were downloaded from the PubChem database (https://pubchem.ncbi.nlm.nih.gov/) in sdf format. All molecular docking between title molecules and core targets were performed using Autodock Vina software (Version 1.5.7) (Trott and Olson, 2010; Morris et al., 2009). The docking results of given substrates at the active site of 6KHM were further examined by the LIGPLOT program (Laskowski and Swindells, 2011), which can generate schematic diagrams of protein-ligand interactions.

Molecular dynamics (MD) simulations were performed to monitor the motions of macromolecules over time (Hildebrand et al., 2019). The protein-ligand complex was simulated using the CHARMM36 force field, implemented with GROMACS 2018.2 software. To generate the topology of the compounds and assign force-field parameters, the CGenFF server was utilized. The protein was placed in a cubic box of dimensions (0.8 × 0.8 × 0.8 Å), filled with TIP3P water molecules, and neutralized with Na^+^ and Cl^−^ ions. The system underwent energy minimization via steepest descent minimization. Subsequently, thermalization was achieved by maintaining the system temperature under the NVT (Number of particles, Volume, and Temperature) ensemble, followed by pressure equilibration under the NPT (Number of particles, Pressure, and Temperature) ensemble. Finally, position restraints were released, and a production MD simulation was run for 100 ns to collect dynamic data (Hou et al., 2023).

## 3 Results and discussion

### 3.1 Orbital analysis (HOMO/LUMO) in various solvents

Frontier molecular orbitals (HOMO-LUMO) are widely used to analyze the stability, responsiveness, and optical/electrical properties of materials (Gopalakrishnan et al., 2014; Singh and Singh, 2015). The energy gap (ΔE) is a critical parameter, as its reduction indicates an increase in chemical reactivity and it is related to the transport of charges in molecules (Yılmaz and Kebiroglu, 2024; Bulut et al., 2025). The estimated ΔE values between HOMO and LUMO are provided in Supplementary Table S1.

**FIGURE 2:**
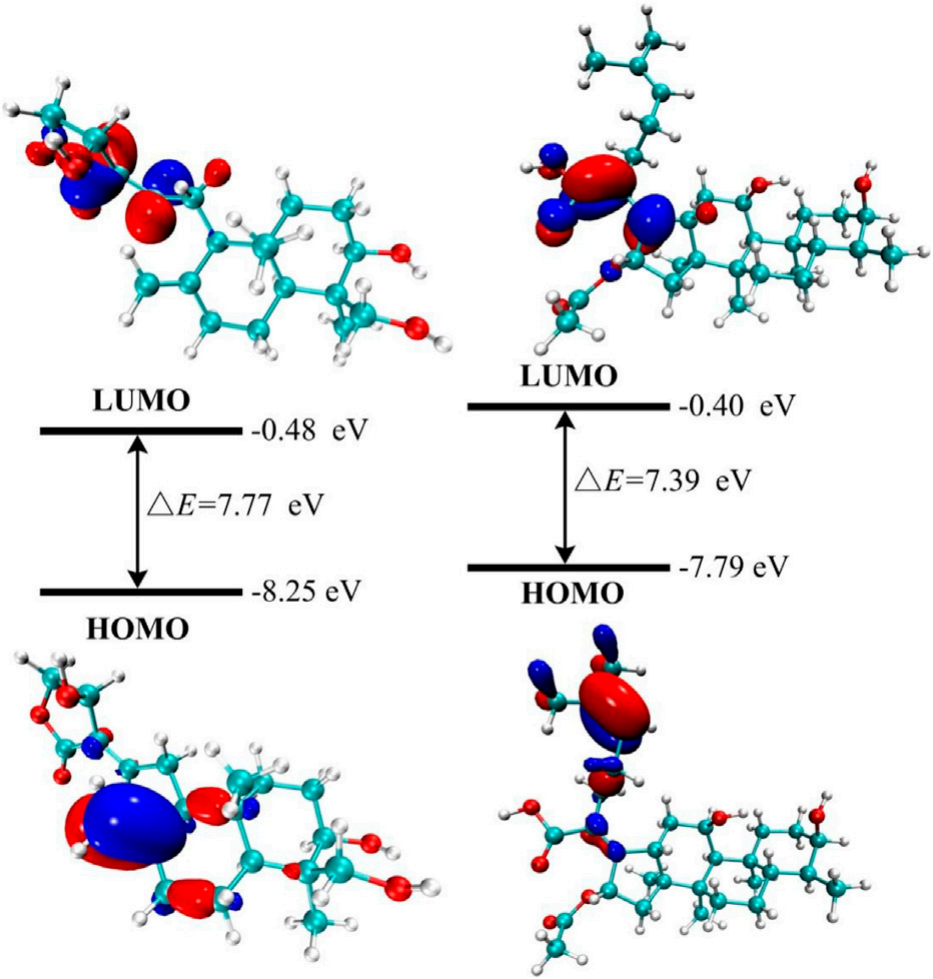
Frontier molecular orbital of the title compounds (AGP: left, FA: right) in gas phase.

The solvent effect is investigated to better understand the chemical characteristics of the molecule. The molecule’s stability is indicated by the negative energies of the HOMO and LUMO orbitals, as well as the surrounding orbitals, as shown in Supplementary Table S1. In the gas phase, as depicted in Figure 2, FA exhibits a lower energy gap (ΔE), which positively correlates with its molecular more reactive (Jaque and Toro-Labbé, 2002; Gopalakrishnan et al., 2014). Furthermore, theoretical research suggests that FA may exhibit enhanced reactive due to its lower value in the HOMO-LUMO energy gap (Bredas, 2014). The electron cloud is primarily localized at the HOMO orbital on the carbon-carbon bond near the five-membered ring. Additional data are provided in Supplementary Table S1.

### 3.2 Analysis of molecular electrostatic potential and electron density analysis in various solvents

**FIGURE 3:**
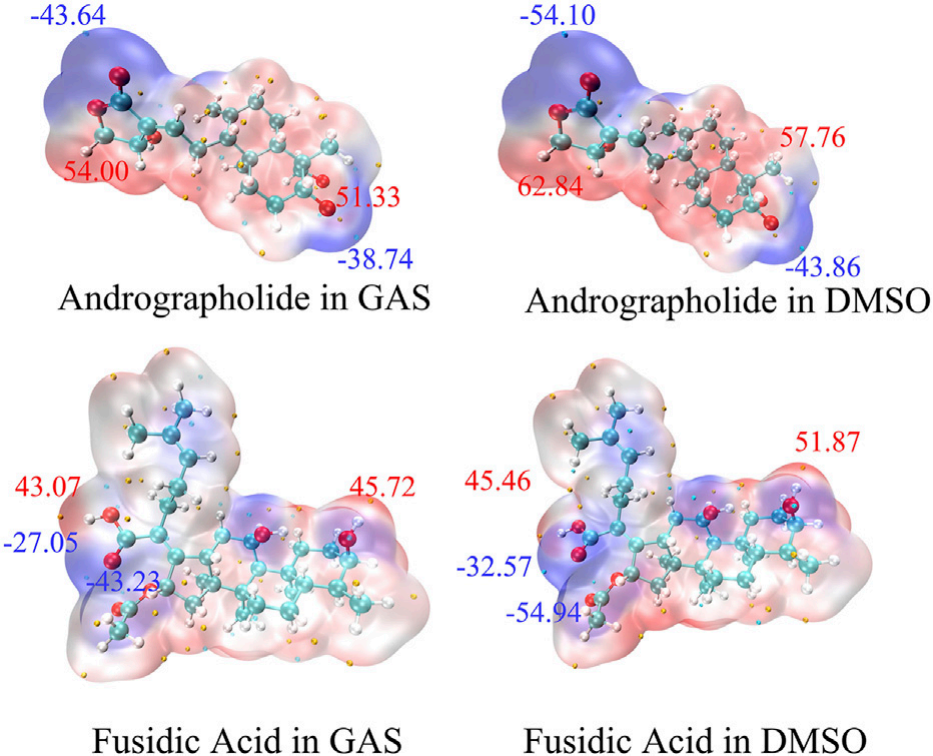
The Molecular Electrostatic Potential (MEP) of AGP and FA in different solvents. The noticeable surface local minima and maximum of the ESP are shown as purple and red spheres, respectively. (Unit:kcal/mol).

The analysis of Molecular Electrostatic Potential (MEP) surfaces plays a pivotal role in identifying electrophilic (negative potential) and nucleophilic (positive potential) regions, which are critical for understanding hydrogen bonding interactions and chemical reactivity (Lu and Chen, 2012b). These surfaces further provide quantitative insights into molecular dimensions, morphological features, reactive site distribution, and charge density patterns. To model the MEP surfaces of the target molecules, we employed the M062X/def2TZVP theoretical methods (Hill, 2013). Under both gas-phase and dimethyl sulfoxide (DMSO) solvent conditions. As illustrated in Figure 3, the resultant MEP maps employ a continuous color gradient to visualize electrostatic potential distributions. Specifically, nucleophilic regions (negative electrostatic potential) are represented by purple isosurfaces, while electrophilic zones (positive electrostatic potential) are depicted in red. Notably, the peripheral purple regions surrounding the oxygen atoms in the target molecules indicate pronounced nucleophilic character. This spatial correlation suggests that oxygen-centered nucleophilic sites may serve as reliable predictors of molecular reactivity patterns.

At the DFT-M06-2x/def2tzvp level, the electrostatic potential (ESP) distribution is quantitatively analyzed and mapped onto the solvent-accessible surface (SAS) in Figure 3. Regions displaying enhanced reactivity in DMSO solution were identified through elevated MEP values (ΔMEP > 10 kcal/mol vs. gas phase). The AGP oxygen moiety exhibited the most negative SAS-ESP value (−54.10 kcal/mol), while the FA structure demonstrated even greater polarization (−54.94 kcal/mol) in DMSO. This systematic analysis demonstrates that solvation effects amplify electrophilic reactivity. Comparative ESP mapping revealed that target compounds have no statistically significant differences in nucleophilic/electrophilic across the studied solvent systems, suggesting similar reactive site accessibility under varying dielectric conditions.

**FIGURE 4:**
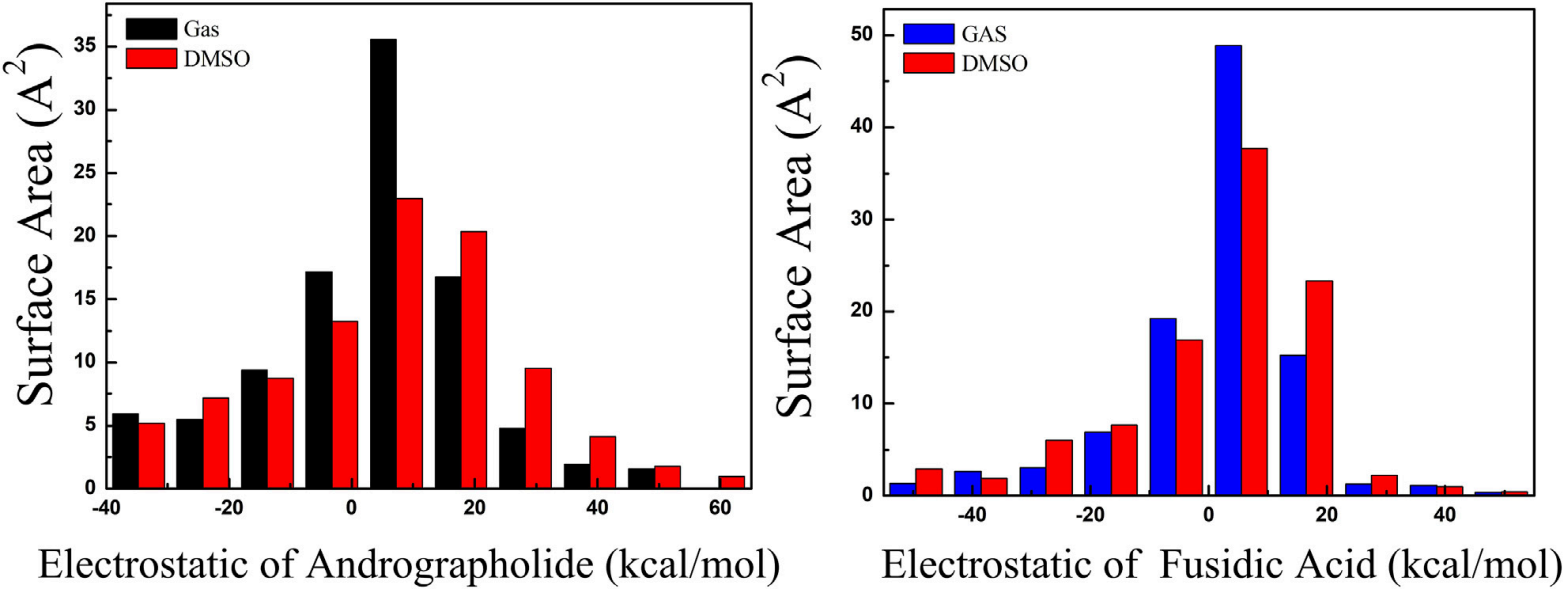
Surface area inside each ESP range on the vdW surface of target compounds. As black, red and blue colors represented different solvent, respectively. (Unit:kcal/mol).

The surface areas of various ESP ranges in different solvents are illustrated in Figure 4, with values ranging from −60 to 60 kcal/mol, represented by distinct colors. The ESP values for the title compounds are predominantly positive, centered around 10 kcal/mol. Through a comparative analysis of the AGP and FA structures in Figure 4, it is evident that the ESP distribution for AGP exhibits a significantly broader range compared to FA. The variance of both positive and negative ESP areas was calculated. For AGP, the values are 258.33 and 383.98 (kcal/mol)^2^, respectively, while for FA, the corresponding values are 222.6 and 275.79 (kcal/mol)^2^. The notable difference in these values indicates that the ESP distribution on the van der Waals surface of AGP exhibits significantly greater fluctuations compared to FA.

### 3.3 Molecular docking and molecular dynamics

#### 3.3.1 Interactions between AGP and FA with _CA_lipase

*C. acnes* lipase is an essential protein in infection, it damages host tissue by hydrolyzing triglycerides into free fatty acids, which in turn induces low-grade inflammation and promotes bacterial colonization (Nakatsuji et al., 2009). _CA_lipase (PDB code is 6KHM) is one of the lipases which is containing the catalytic triad Ser-Asp-His. From the past researches (Yu et al., 2022), we know that catalytic triad of _CA_lipase could be one of the traditional binding sites for molecule inhibitors. However, through the screening of Autodock Vina software, it is determined that in addition to the Ser-Asp-His enzyme activity center, there may be another better binding pocket during the screening results.

**FIGURE 5:**
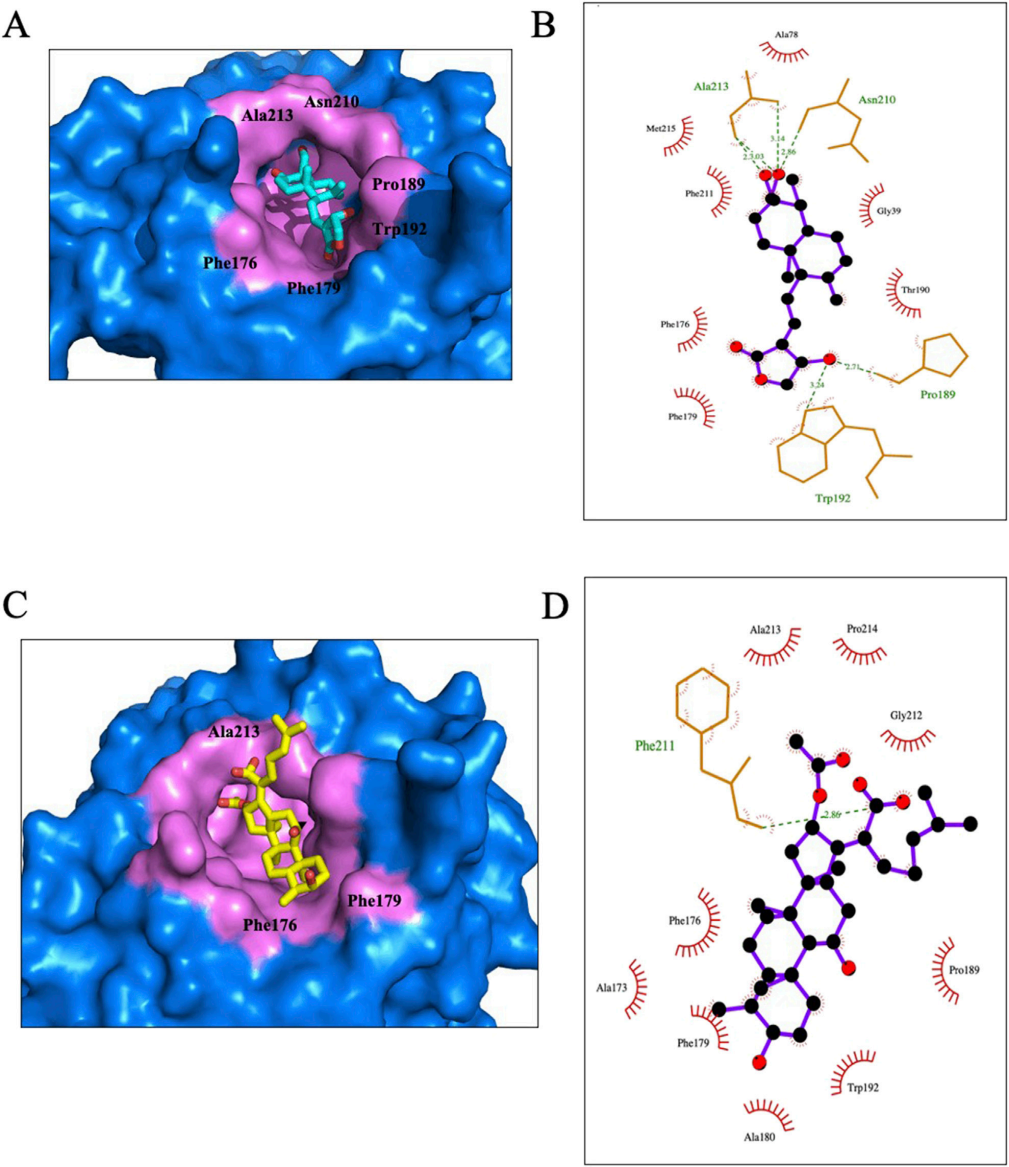
Molecular interaction of AGP and FA with _CA_lipase, respectively. Cross-section of ligands docked in binding pocket of _CA_lipase **(A,C)**, Ligplot **(B,D)** showing residues involved in interactions between AGP and FA.

As expected, AGP and FA fitted very well into the screened binding pocket, even so, there is still some subtle difference exists between the two complexes. In Figure 5, we can see AGP and FA relied on the gorge of the pocket, and there are some solvent hydrophobic interactions with the surrounding residues. The co-interact residues are Phe176 and Phe179. Besides, AGP is interact with Gly39, Ala78, Thr190, Phe211 and Met215 which are participated in forming a hydrophobic pocket to accommodate molecular (Figure 5A). In addition to the hydrophobic interaction, the Ligplot 2D diagram analysis revealed that AGP has some unique hydrogen bond interactions comparing with FA. From Figure 5, AGP carbonyl oxygen forms hydrogen bonds with the main chain amides of Pro189, Asn210 and Ala213, and has some strong hydrogen bond interaction the bulky side chain of Trp192.

In the above mentioned residues, Phe176, Phe179, Trp192 and Phe211, Which all possess bulky side chains, are confirmed to participate in the activities of _CA_lipase (Kim et al., 2020),it further proved that AGP might be a potential and effective inhibitor of _CA_lipase.

#### 3.3.2 Molecular dynamics between AGP and FA

**FIGURE 6:**
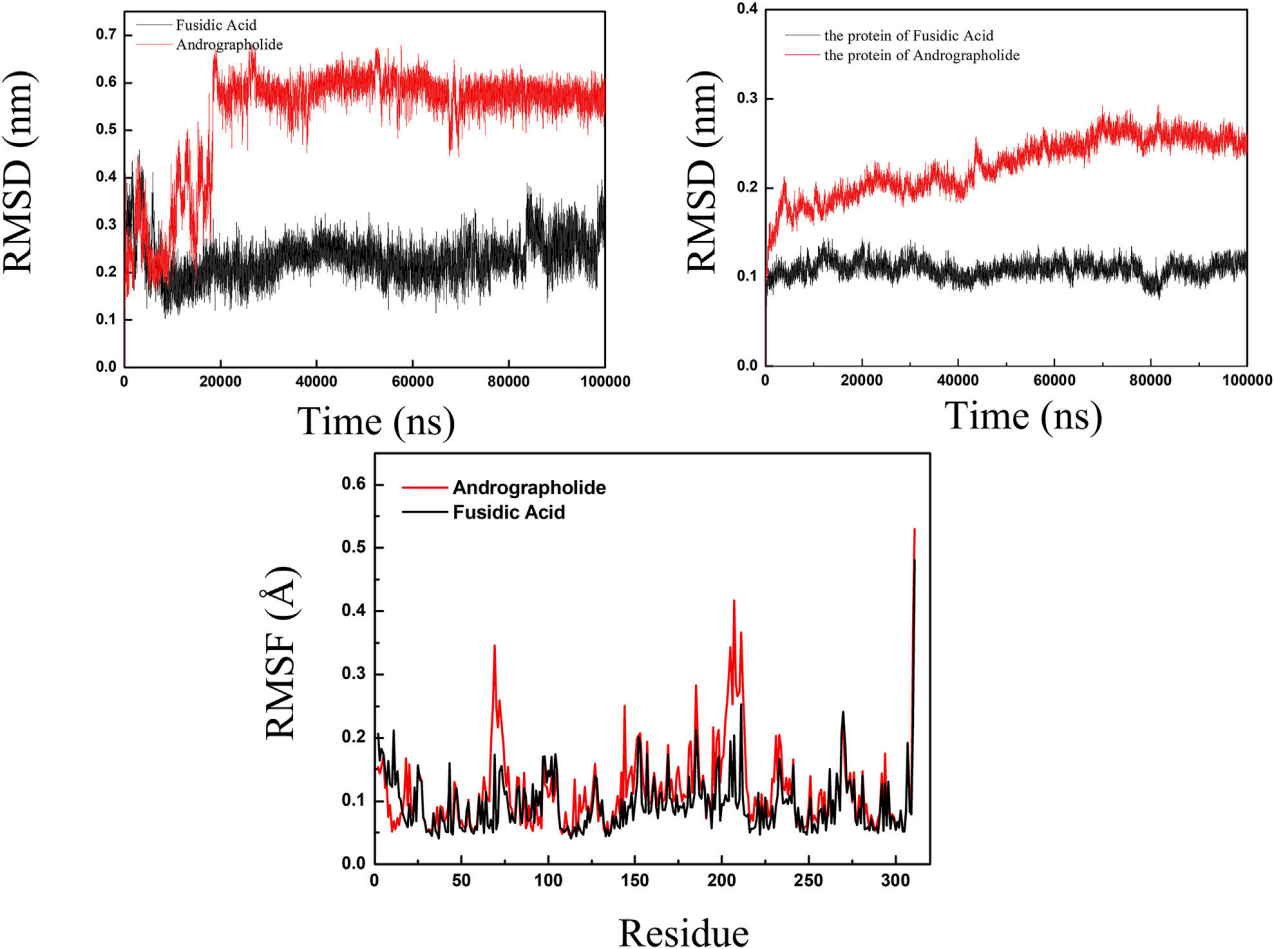
The specific information of RMSD plot and RMSF polt. (red line representedAGP-6KHW and black line represented FA-6KHW).

RMSD analysis for evaluating structural stability of protein-ligand complexes are shown in Figure 6, following established molecular dynamics protocols (Rudnev et al., 2022). Comparative simulations of AGP-6KHW and FA-6KHW complexes were conducted over a 100 ns trajectory. Panel A demonstrates distinct ligand stabilization patterns: AGP exhibits an initial RMSD increment (0–20 ns) followed by equilibration, while FA achieves conformational stability earlier (post-10 ns) with sustained equilibrium throughout 10–90 ns. The protein backbone RMSD in Panel B reveals concomitant stabilization, with both complexes maintaining marginal fluctuations in the terminal 30 ns. This convergent ligand-protein stabilization profile confirms the systems’ structural integrity, evidenced by sustained compact folding geometries throughout the simulation period. RMSF was used to calculate the root mean square fluctuations of atomic positions in trajectories (Thangavel and Albratty, 2023). The results indicated that there was little fluctuation in the atomic positions of amino acids around the docking site of 6KHW protein from Supplementary Figure S1. The molecular dynamics results indicated that in a 100 ns simulation, the binding of AGP-6KHW and FA-6KHW complexes were stable and persistent.

### 3.4 The independent gradient model in Hirshfeld partition of molecular density analysis

The analysis of intra-fragment (δg_intra) and inter-fragment (δg_inter) interactions commonly employs the Independent Gradient Model based on Hirshfeld partition (IGMH) methodology (Cao et al., 2017). In the present study, IGMH analysis was conducted to elucidate protein-compound interaction mechanisms. Within this framework, the δg_inter parameter quantitatively characterizes solvent-compound interaction components in computational simulations. These intermolecular interactions are visually represented through color-coded δg_inter isosurfaces mapped by the sign (I2)r function, which encodes both interaction strength and nature (Wang et al., 2022). The established Blue-Green-Red (BGR) chromatic scheme associates specific interaction types with distinct color domains: blue regions correspond to energetically favorable hydrogen-bonding interactions, while green areas indicate weaker van der Waals (vdW) forces and other less-stabilizing interactions.

**FIGURE 7:**
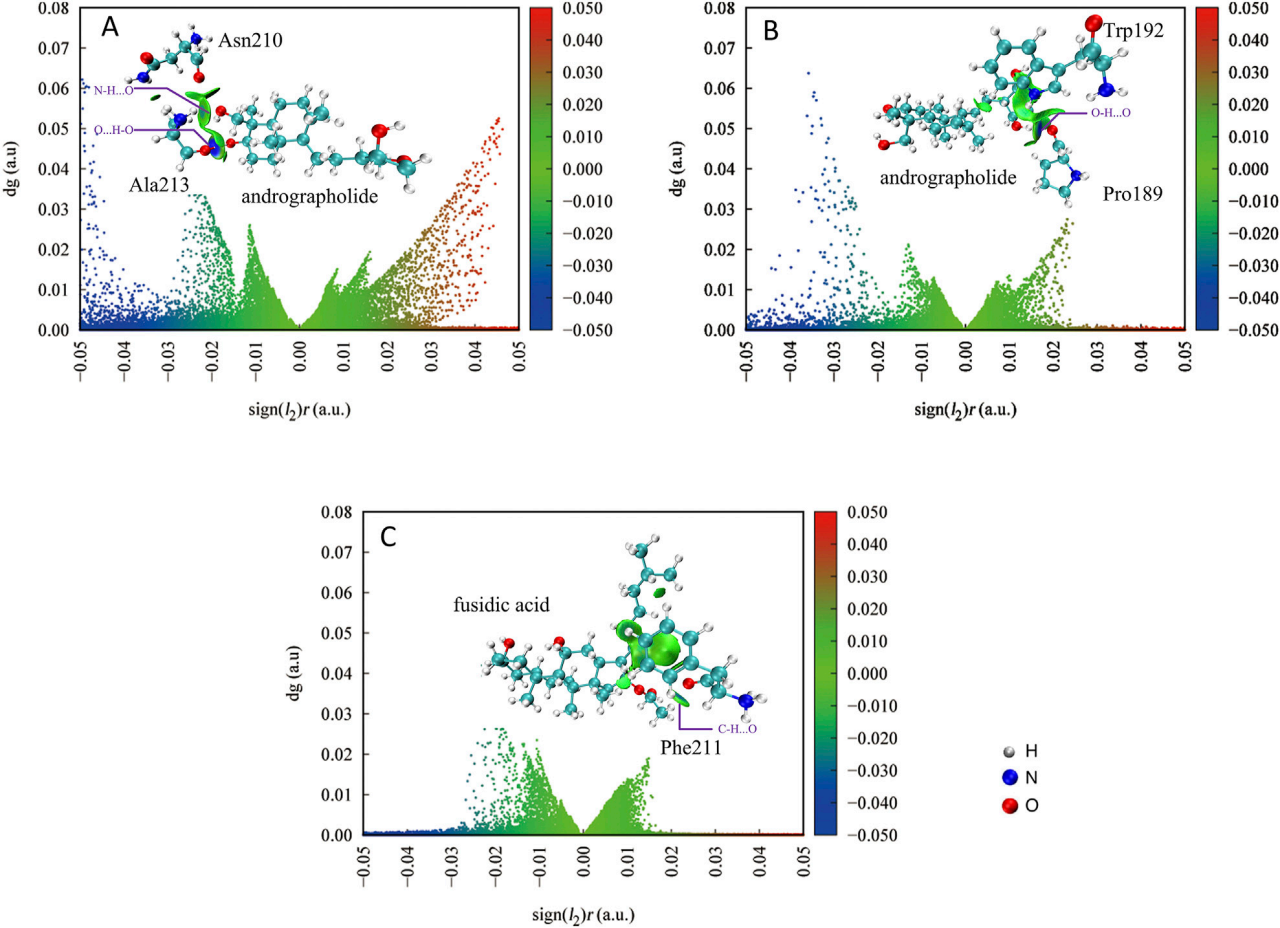
The IGMH scatter plot quantitatively delineates specific non-covalent interaction domains between the target compound and protein residues, with the characteristic δg_inter isosurface threshold set at 0.005 atomic units by Multiwfn 3.8 software. AGP compound and associated protein residues **(A,B)**; FA compound and associated protein residues **(C)**.

There are some weak interactions between AGP and associated proteins in Figures 7A,B, as evidenced by interaction energy values exceeding −0.05 a.u. in both panels. Hydrogen bonding interactions are observed in both cases. In Figure 7A, two distinct hydrogen bonds are formed between protein residue Ala213 and AGP: an O···H-O interaction and an N-H···O interaction. The significantly deeper blue isosurface associated with the O···H-O bond indicates its stronger interaction energy compared to the N-H···O counterpart. Notably, the interaction between Asn210 and AGP’s C-H···N group displays minimal green isosurfaces, suggesting a weak interaction dominated by dispersion forces with low electron density distribution in the bonding region.

Figure 7B demonstrates that Protein189 forms an O···H-O hydrogen bond with AGP, while other interfacial regions primarily exhibit weak interactions represented by green isosurfaces. This pattern indicates that the majority of these secondary interactions are likely mediated through van der Waals forces rather than specific hydrogen bonding.

For comparative analysis, Figure 7C presents the IGM-H plot of control FA interacting with Phe211. The absence of weak hydrogen bonding is not evident, with only marginal C-H···O interactions. The interaction profile predominantly features dispersion-driven contacts, further confirming the weaker binding characteristics of the control compound. In summary, AGP demonstrates superior binding characteristics through its ability to form multiple specific hydrogen bonds (particularly the strong O···H-O interaction) complemented by secondary weak interactions. This contrasts markedly with the FA control system that relies primarily on non-specific dispersion forces, highlighting AGP’s enhanced molecular interaction capabilities.

## 4 Conclusion

*C. acnes* is a bacterium with dual roles as both a commensal organism and an opportunistic pathogen. While it plays a protective role on the skin, under certain conditions, it contributes to the pathogenesis of acne and other infections. Treatment strategies for *C. acnes* related conditions vary depending on the severity and type of infection, but typically involve a combination of antimicrobial, anti-inflammatory and sebum-reducing therapies.

According to the past researches, lipases of *C. acnes* hydrolyze sebum triglycerides into free fatty acids and glycerol, which can provide a nutrient source for bacterial growth. And the crystal structure of lipase has shown it features a hydrophobic lid that covers its active site, which opens to interact with lipid substrates.

AGP, a labdane diterpenoid lactone extracted from *Andrographis paniculata*, has attracted growing interest due to its broad-spectrum pharmacological activities, including anti-inflammatory, antibacterial. Despite this, its specific interaction with _CA_lipase has not been thoroughly investigated.

In this work, we utilized computational methods based on molecular structure obtained the chemistry parameters and molecular dynamics results. We compared AGP and FA, which works well against *C. acnes s* in a clinical setting, and reported that AGP has a certain effective interaction with _CA_lipase than FA. Through our computational analyses, we observed that AGP exhibits a notably stronger and more specific interaction with _CA_lipase compared to FA, suggesting a potentially more effective inhibitory mechanism.

These findings position AGP as a promising candidate for further development as a therapeutic agent targeting C. acnes. Importantly, our results open the door to more detailed mechanistic studies. Future work we could investigate the precise mechanism by whether it inhibits the growth of *C. acnes* directly or influences biofilm formation of *C. acnes* at the genetic level. This study also sets the stage for structure activity relationship analyses and the design of AGP derivatives with enhanced binding affinity and specificity for _*CA*_lipase. The insights gained from this work contribute to a growing body of research exploring small-molecule inhibitors against bacterial lipases and support the use of *in silico* approaches as powerful tools in drug discovery and development. It should be emphasized that while our computational results are promising, additional research and comprehensive clinical studies are still required to completely evaluate their safety, effectiveness, and potential applications in management.

## Data Availability

The original contributions presented in the study are included in the article/Supplementary Material, further inquiries can be directed to the corresponding author.
