# Supplementary material for: Computational methodology and molecular dynamics analysis of andrographolide bioactivity against Cutibacterium acnes
Source: Front Chem. 2025 Jun 11;13:1627758. doi: 10.3389/fchem.2025.1627758 (PMC12187781; doi:10.3389/fchem.2025.1627758)
Supplement: Supplementary file 1 [file Supplementaryfile1.docx]

# *Supplementary Material*

# Computational methodology and Molecular Dynamics analysis of andrographolide bioactivity against Cutibacterium acnes

Min Lin ^a^†, Lihui He ^b^†, Guodong Ye ^c^, Xiaotian Zhao ^b^ *

^a^ *Department of Dermatology, Chengdu Second Peoples Hospital, Chengdu, 610017, P.R. China*

^b^ *Department of Pharmacy, Chengdu Second Peoples Hospital, Chengdu, 610017, P.R. China*

^c^ *The Fifth Affi1iated Hospita1, Guangdong Province & NMPA & State Key Laboratory, Schoo1 of Pharmaceutica1 Sciences, Guangzhou Medical Medica1 University, Guangzhou, 511436, China.*

***** Correspondence: cshpharmcyzxt@foxmail.com

† *These authors have contributed equally to this work*

### **Cartesian coordinates the optimized structure** (X,Y,Z)

#### AGP

| Molecular in gas phase |  |  |  |  |
| --- | --- | --- | --- | --- |
|  |  |  |  |  |
| \| O \|  \| 6.217554 \| -0.22341 \| -0.44054 \| \| --- \| --- \| --- \| --- \| --- \| \| C \|  \| 5.105424 \| 0.205564 \| -1.09128 \| \| C \|  \| 3.91522 \| -0.34382 \| -0.39575 \| \| C \|  \| 4.377156 \| -1.07028 \| 0.829431 \| \| C \|  \| 5.890961 \| -1.16923 \| 0.574982 \| \| O \|  \| 5.155853 \| 0.912606 \| -2.04994 \| \| O \|  \| 4.054777 \| -0.28251 \| 1.965984 \| \| C \|  \| 2.675831 \| -0.13553 \| -0.81959 \| \| C \|  \| 1.438225 \| -0.6614 \| -0.16387 \| \| C \|  \| 0.24237 \| 0.277562 \| -0.34418 \| \| C \|  \| 0.448038 \| 1.651464 \| 0.24771 \| \| C \|  \| -0.60709 \| 2.630996 \| -0.18042 \| \| C \|  \| -2.00668 \| 2.106243 \| 0.153108 \| \| C \|  \| -2.22023 \| 0.68465 \| -0.37947 \| \| C \|  \| -3.70153 \| 0.206463 \| -0.29774 \| \| C \|  \| -3.7769 \| -1.18199 \| -0.9729 \| \| C \|  \| -2.79261 \| -2.17435 \| -0.37413 \| \| C \|  \| -1.35635 \| -1.68006 \| -0.49524 \| \| C \|  \| -1.12814 \| -0.30163 \| 0.145211 \| \| C \|  \| 1.414336 \| 1.968682 \| 1.101548 \| \| C \|  \| -1.09792 \| -0.4513 \| 1.675377 \| \| C \|  \| -4.59535 \| 1.168624 \| -1.09549 \| \| C \|  \| -4.20982 \| 0.172189 \| 1.147007 \| \| O \|  \| -5.61445 \| -0.09212 \| 1.14074 \| \| O \|  \| -5.07944 \| -1.72895 \| -1.00796 \| \| H \|  \| 3.929084 \| -2.06365 \| 0.921314 \| \| H \|  \| 6.172973 \| -2.16354 \| 0.224065 \| \| H \|  \| 6.462884 \| -0.92119 \| 1.468351 \| \| H \|  \| 4.328204 \| -0.74947 \| 2.761913 \| \| H \|  \| 2.560133 \| 0.452241 \| -1.72817 \| \| H \|  \| 1.205745 \| -1.61839 \| -0.64079 \| \| H \|  \| 1.642389 \| -0.86754 \| 0.887968 \| \| H \|  \| 0.128534 \| 0.415079 \| -1.42892 \| \| H \|  \| -0.44087 \| 3.601965 \| 0.287209 \| \| H \|  \| -0.53796 \| 2.773667 \| -1.265 \| \| H \|  \| -2.14222 \| 2.141376 \| 1.236078 \| \| H \|  \| -2.74944 \| 2.778279 \| -0.27694 \| \| H \|  \| -2.03461 \| 0.738445 \| -1.46179 \| \| H \|  \| -3.49168 \| -1.02559 \| -2.0218 \| \| H \|  \| -3.05865 \| -2.37266 \| 0.668056 \| \| H \|  \| -2.90906 \| -3.12251 \| -0.90141 \| \| H \|  \| -0.68321 \| -2.41292 \| -0.04441 \| \| H \|  \| -1.09514 \| -1.62131 \| -1.55908 \| \| H \|  \| 1.478317 \| 2.973535 \| 1.50263 \| \| H \|  \| 2.174322 \| 1.267842 \| 1.423499 \| \| H \|  \| -1.3092 \| 0.481017 \| 2.196837 \| \| H \|  \| -1.80487 \| -1.20379 \| 2.019874 \| \| H \|  \| -0.11059 \| -0.77596 \| 2.005308 \| \| H \|  \| -4.12457 \| 1.414721 \| -2.04969 \| \| H \|  \| -5.55888 \| 0.707344 \| -1.29991 \| \| H \|  \| -4.78156 \| 2.095502 \| -0.55276 \| \| H \|  \| -4.02474 \| 1.14298 \| 1.616128 \| \| H \|  \| -3.70174 \| -0.59238 \| 1.733755 \| \| H \|  \| -5.93002 \| -0.13836 \| 2.046604 \| \| H \|  \| -5.55747 \| -1.4369 \| -0.22113 \| |  |  |  |  |
| Molecular in DMSO |  |  |  |  |
| \| O \|  \| 6.204753 \| -0.30841 \| -0.4688 \| \| --- \| --- \| --- \| --- \| --- \| \| C \|  \| 5.098763 \| 0.127953 \| -1.10265 \| \| C \|  \| 3.907141 \| -0.34637 \| -0.36405 \| \| C \|  \| 4.382305 \| -0.97233 \| 0.9127 \| \| C \|  \| 5.857531 \| -1.2204 \| 0.591137 \| \| O \|  \| 5.16031 \| 0.802109 \| -2.09545 \| \| O \|  \| 4.205102 \| -0.0248 \| 1.958885 \| \| C \|  \| 2.664827 \| -0.18825 \| -0.80745 \| \| C \|  \| 1.431272 \| -0.67298 \| -0.11924 \| \| C \|  \| 0.238522 \| 0.26 \| -0.34681 \| \| C \|  \| 0.43962 \| 1.65742 \| 0.188054 \| \| C \|  \| -0.61398 \| 2.619012 \| -0.28148 \| \| C \|  \| -2.01476 \| 2.106899 \| 0.06706 \| \| C \|  \| -2.22302 \| 0.665611 \| -0.4097 \| \| C \|  \| -3.70387 \| 0.190001 \| -0.30911 \| \| C \|  \| -3.77677 \| -1.22351 \| -0.92744 \| \| C \|  \| -2.79258 \| -2.19039 \| -0.29161 \| \| C \|  \| -1.35661 \| -1.69857 \| -0.42687 \| \| C \|  \| -1.13496 \| -0.29701 \| 0.16226 \| \| C \|  \| 1.402382 \| 2.010845 \| 1.03534 \| \| C \|  \| -1.11455 \| -0.38398 \| 1.696854 \| \| C \|  \| -4.58963 \| 1.122223 \| -1.14903 \| \| C \|  \| -4.2167 \| 0.211458 \| 1.133934 \| \| O \|  \| -5.62925 \| -0.0425 \| 1.135855 \| \| O \|  \| -5.08975 \| -1.77201 \| -0.91831 \| \| H \|  \| 3.869996 \| -1.90627 \| 1.149076 \| \| H \|  \| 6.020135 \| -2.23756 \| 0.233644 \| \| H \|  \| 6.508138 \| -1.01701 \| 1.439447 \| \| H \|  \| 4.497257 \| -0.4304 \| 2.785394 \| \| H \|  \| 2.533597 \| 0.307361 \| -1.76791 \| \| H \|  \| 1.196967 \| -1.64855 \| -0.55612 \| \| H \|  \| 1.634769 \| -0.83924 \| 0.939636 \| \| H \|  \| 0.128363 \| 0.35324 \| -1.43545 \| \| H \|  \| -0.45032 \| 3.608094 \| 0.149121 \| \| H \|  \| -0.54069 \| 2.713195 \| -1.37097 \| \| H \|  \| -2.16006 \| 2.184383 \| 1.1465 \| \| H \|  \| -2.75128 \| 2.763539 \| -0.39643 \| \| H \|  \| -2.03262 \| 0.672761 \| -1.49193 \| \| H \|  \| -3.50215 \| -1.11246 \| -1.98434 \| \| H \|  \| -3.06017 \| -2.34821 \| 0.757096 \| \| H \|  \| -2.89744 \| -3.15818 \| -0.78706 \| \| H \|  \| -0.68572 \| -2.41316 \| 0.055121 \| \| H \|  \| -1.09082 \| -1.67704 \| -1.49058 \| \| H \|  \| 1.462709 \| 3.030648 \| 1.401651 \| \| H \|  \| 2.156911 \| 1.318586 \| 1.390642 \| \| H \|  \| -1.33916 \| 0.567262 \| 2.179388 \| \| H \|  \| -1.82262 \| -1.12278 \| 2.068931 \| \| H \|  \| -0.12854 \| -0.69535 \| 2.046742 \| \| H \|  \| -4.11781 \| 1.320329 \| -2.1145 \| \| H \|  \| -5.56093 \| 0.665737 \| -1.33264 \| \| H \|  \| -4.76422 \| 2.075742 \| -0.64946 \| \| H \|  \| -4.02892 \| 1.194732 \| 1.573416 \| \| H \|  \| -3.72338 \| -0.53772 \| 1.750978 \| \| H \|  \| -5.91931 \| -0.12478 \| 2.051255 \| \| H \|  \| -5.55161 \| -1.40781 \| -0.14958 \| |  |  |  |  |
|  |  |  |  |  |
|  |  |  |  |  |
|  |  |  |  |  |
|  |  |  |  |  |
|  |  |  |  |  |
|  |  |  |  |  |

#### FA

Molecular in gas phase

| \| C \|  \| 2.088531 \| -2.88135 \| -0.948 \| \| --- \| --- \| --- \| --- \| --- \| \| C \|  \| 1.581825 \| -1.56838 \| -0.31309 \| \| C \|  \| 0.017254 \| -1.6046 \| -0.44181 \| \| C \|  \| 2.144574 \| -0.29605 \| -1.04428 \| \| C \|  \| 2.045082 \| -1.56089 \| 1.156444 \| \| C \|  \| -0.40347 \| -1.9669 \| -1.88186 \| \| C \|  \| -0.65839 \| -0.2702 \| -0.05049 \| \| C \|  \| -0.69291 \| -2.56171 \| 0.529981 \| \| C \|  \| 3.690176 \| -0.07397 \| -0.84989 \| \| C \|  \| 1.32538 \| 0.997408 \| -0.79243 \| \| C \|  \| 3.556166 \| -1.39228 \| 1.327945 \| \| C \|  \| -2.12152 \| -0.63678 \| 0.12956 \| \| C \|  \| -0.18123 \| 0.842621 \| -0.96144 \| \| C \|  \| -2.15596 \| -2.11059 \| 0.533411 \| \| C \|  \| 4.479289 \| -1.13419 \| -1.6525 \| \| C \|  \| 4.06679 \| -0.12341 \| 0.656041 \| \| C \|  \| 4.114026 \| 1.300453 \| -1.42245 \| \| O \|  \| 1.478256 \| 1.561905 \| 0.508137 \| \| C \|  \| -3.21351 \| 0.132725 \| 0.012063 \| \| O \|  \| -2.91833 \| -2.81913 \| -0.44532 \| \| C \|  \| 5.562154 \| 0.153291 \| 0.880437 \| \| C \|  \| 5.575755 \| 1.655165 \| -1.1499 \| \| C \|  \| -3.19665 \| 1.566674 \| -0.45772 \| \| C \|  \| -4.5328 \| -0.39707 \| 0.469807 \| \| C \|  \| -3.66396 \| -3.89786 \| -0.12468 \| \| C \|  \| 5.970234 \| 0.050428 \| 2.347588 \| \| C \|  \| 5.898777 \| 1.542555 \| 0.333812 \| \| C \|  \| -2.86205 \| 2.541223 \| 0.683639 \| \| O \|  \| -5.55386 \| 0.439269 \| 0.18679 \| \| O \|  \| -4.74073 \| -1.41924 \| 1.074033 \| \| C \|  \| -3.627 \| -4.39731 \| 1.297065 \| \| O \|  \| -4.31003 \| -4.41944 \| -0.9857 \| \| O \|  \| 5.135321 \| 2.487311 \| 1.086744 \| \| C \|  \| -2.80454 \| 3.966466 \| 0.21066 \| \| C \|  \| -3.8502 \| 4.767841 \| 0.018324 \| \| C \|  \| -3.68282 \| 6.172909 \| -0.48425 \| \| C \|  \| -5.27231 \| 4.356808 \| 0.274119 \| \| H \|  \| 2.092359 \| -2.84291 \| -2.03693 \| \| H \|  \| 3.087072 \| -3.14776 \| -0.62093 \| \| H \|  \| 1.435871 \| -3.70297 \| -0.64816 \| \| H \|  \| 2.01375 \| -0.46704 \| -2.11988 \| \| H \|  \| 1.549869 \| -0.75293 \| 1.701262 \| \| H \|  \| 1.754708 \| -2.50186 \| 1.628573 \| \| H \|  \| 0.193909 \| -1.44481 \| -2.62867 \| \| H \|  \| -0.30051 \| -3.03581 \| -2.06307 \| \| H \|  \| -1.44983 \| -1.71643 \| -2.05803 \| \| H \|  \| -0.31918 \| -0.01287 \| 0.963423 \| \| H \|  \| -0.30092 \| -2.43582 \| 1.539537 \| \| H \|  \| -0.61159 \| -3.61482 \| 0.258174 \| \| H \|  \| 1.644073 \| 1.729662 \| -1.53806 \| \| H \|  \| 3.765524 \| -1.34059 \| 2.397276 \| \| H \|  \| 4.094814 \| -2.27105 \| 0.96219 \| \| H \|  \| -0.43461 \| 0.633897 \| -2.00259 \| \| H \|  \| -0.60302 \| 1.809619 \| -0.69775 \| \| H \|  \| -2.61695 \| -2.21857 \| 1.510189 \| \| H \|  \| 5.469239 \| -0.77427 \| -1.92352 \| \| H \|  \| 4.630905 \| -2.0614 \| -1.1085 \| \| H \|  \| 3.961691 \| -1.37418 \| -2.58211 \| \| H \|  \| 3.552524 \| 0.69099 \| 1.164948 \| \| H \|  \| 3.914931 \| 1.322199 \| -2.49886 \| \| H \|  \| 3.524078 \| 2.104748 \| -0.98436 \| \| H \|  \| 2.34673 \| 1.967497 \| 0.593667 \| \| H \|  \| 6.156392 \| -0.56597 \| 0.305848 \| \| H \|  \| 6.265666 \| 1.021215 \| -1.70886 \| \| H \|  \| 5.767413 \| 2.67886 \| -1.48568 \| \| H \|  \| -2.48721 \| 1.688278 \| -1.27089 \| \| H \|  \| -4.16934 \| 1.831408 \| -0.86603 \| \| H \|  \| 5.313952 \| 0.657741 \| 2.97265 \| \| H \|  \| 6.99097 \| 0.412455 \| 2.484997 \| \| H \|  \| 5.933278 \| -0.97841 \| 2.703463 \| \| H \|  \| 6.966365 \| 1.733819 \| 0.495186 \| \| H \|  \| -3.60642 \| 2.420544 \| 1.474863 \| \| H \|  \| -1.90056 \| 2.259647 \| 1.121063 \| \| H \|  \| -6.35128 \| 0.014945 \| 0.533944 \| \| H \|  \| -4.12834 \| -3.67982 \| 1.946588 \| \| H \|  \| -4.15309 \| -5.34598 \| 1.325863 \| \| H \|  \| -2.6038 \| -4.52865 \| 1.650103 \| \| H \|  \| 5.43718 \| 3.37289 \| 0.866472 \| \| H \|  \| -1.81635 \| 4.354603 \| -0.02225 \| \| H \|  \| -2.63483 \| 6.41939 \| -0.64894 \| \| H \|  \| -4.22437 \| 6.314228 \| -1.42351 \| \| H \|  \| -4.09885 \| 6.88902 \| 0.229725 \| \| H \|  \| -5.3653 \| 3.327707 \| 0.615599 \| \| H \|  \| -5.73021 \| 5.014114 \| 1.018463 \| \| H \|  \| -5.86199 \| 4.461785 \| -0.64074 \| |  |  |  |  |
| --- | --- | --- | --- | --- | --- | --- | --- | --- | --- | --- | --- | --- | --- | --- | --- | --- | --- | --- | --- | --- | --- | --- | --- | --- | --- | --- | --- | --- | --- | --- | --- | --- | --- | --- | --- | --- | --- | --- | --- | --- | --- | --- | --- | --- | --- | --- | --- | --- | --- | --- | --- | --- | --- | --- | --- | --- | --- | --- | --- | --- | --- | --- | --- | --- | --- | --- | --- | --- | --- | --- | --- | --- | --- | --- | --- | --- | --- | --- | --- | --- | --- | --- | --- | --- | --- | --- | --- | --- | --- | --- | --- | --- | --- | --- | --- | --- | --- | --- | --- | --- | --- | --- | --- | --- | --- | --- | --- | --- | --- | --- | --- | --- | --- | --- | --- | --- | --- | --- | --- | --- | --- | --- | --- | --- | --- | --- | --- | --- | --- | --- | --- | --- | --- | --- | --- | --- | --- | --- | --- | --- | --- | --- | --- | --- | --- | --- | --- | --- | --- | --- | --- | --- | --- | --- | --- | --- | --- | --- | --- | --- | --- | --- | --- | --- | --- | --- | --- | --- | --- | --- | --- | --- | --- | --- | --- | --- | --- | --- | --- | --- | --- | --- | --- | --- | --- | --- | --- | --- | --- | --- | --- | --- | --- | --- | --- | --- | --- | --- | --- | --- | --- | --- | --- | --- | --- | --- | --- | --- | --- | --- | --- | --- | --- | --- | --- | --- | --- | --- | --- | --- | --- | --- | --- | --- | --- | --- | --- | --- | --- | --- | --- | --- | --- | --- | --- | --- | --- | --- | --- | --- | --- | --- | --- | --- | --- | --- | --- | --- | --- | --- | --- | --- | --- | --- | --- | --- | --- | --- | --- | --- | --- | --- | --- | --- | --- | --- | --- | --- | --- | --- | --- | --- | --- | --- | --- | --- | --- | --- | --- | --- | --- | --- | --- | --- | --- | --- | --- | --- | --- | --- | --- | --- | --- | --- | --- | --- | --- | --- | --- | --- | --- | --- | --- | --- | --- | --- | --- | --- | --- | --- | --- | --- | --- | --- | --- | --- | --- | --- | --- | --- | --- | --- | --- | --- | --- | --- | --- | --- | --- | --- | --- | --- | --- | --- | --- | --- | --- | --- | --- | --- | --- | --- | --- | --- | --- | --- | --- | --- | --- | --- | --- | --- | --- | --- | --- | --- | --- | --- | --- | --- | --- | --- | --- | --- | --- | --- | --- | --- | --- | --- | --- | --- | --- | --- | --- | --- | --- | --- | --- | --- | --- | --- | --- | --- | --- | --- | --- | --- | --- | --- | --- | --- | --- | --- | --- | --- | --- | --- | --- | --- | --- | --- | --- | --- | --- | --- | --- | --- | --- | --- | --- | --- | --- | --- | --- | --- | --- | --- | --- | --- | --- | --- | --- | --- | --- | --- | --- | --- | --- |
|  |  |  |  |  |
|  |  |  |  |  |
|  |  |  |  |  |

Molecular in DMSO

| C |  | 2.085098 | -2.85949 | -0.96671 |
| --- | --- | --- | --- | --- |
| C |  | 1.590685 | -1.5489 | -0.31729 |
| C |  | 0.026172 | -1.57725 | -0.43567 |
| C |  | 2.151932 | -0.27192 | -1.0432 |
| C |  | 2.064596 | -1.5564 | 1.148365 |
| C |  | -0.40202 | -1.92095 | -1.87704 |
| C |  | -0.63509 | -0.24053 | -0.02311 |
| C |  | -0.68763 | -2.54781 | 0.521319 |
| C |  | 3.700499 | -0.05917 | -0.85977 |
| C |  | 1.341619 | 1.026235 | -0.78414 |
| C |  | 3.578335 | -1.39676 | 1.30774 |
| C |  | -2.09666 | -0.60278 | 0.133383 |
| C |  | -0.16698 | 0.877158 | -0.9306 |
| C |  | -2.13947 | -2.06453 | 0.572092 |
| C |  | 4.473374 | -1.11714 | -1.67937 |
| C |  | 4.09476 | -0.12675 | 0.641327 |
| C |  | 4.131197 | 1.316388 | -1.42427 |
| O |  | 1.522596 | 1.603989 | 0.512497 |
| C |  | -3.19698 | 0.12984 | -0.06061 |
| O |  | -2.98549 | -2.78483 | -0.34238 |
| C |  | 5.596923 | 0.127007 | 0.847941 |
| C |  | 5.600188 | 1.651438 | -1.16603 |
| C |  | -3.23051 | 1.574374 | -0.50074 |
| C |  | -4.52655 | -0.48376 | 0.245856 |
| C |  | -3.69447 | -3.85327 | 0.053512 |
| C |  | 6.023433 | 0.000318 | 2.307629 |
| C |  | 5.955296 | 1.512504 | 0.306961 |
| C |  | -2.92476 | 2.525485 | 0.668935 |
| O |  | -5.45724 | -0.17302 | -0.66879 |
| O |  | -4.79319 | -1.16359 | 1.206477 |
| C |  | -3.5544 | -4.32773 | 1.470507 |
| O |  | -4.41166 | -4.38562 | -0.75463 |
| O |  | 5.245024 | 2.476118 | 1.092036 |
| C |  | -2.90188 | 3.960665 | 0.227375 |
| C |  | -3.95017 | 4.782321 | 0.156224 |
| C |  | -3.80876 | 6.195195 | -0.33177 |
| C |  | -5.34945 | 4.390703 | 0.533749 |
| H |  | 2.095406 | -2.80486 | -2.05554 |
| H |  | 3.079377 | -3.14283 | -0.6383 |
| H |  | 1.420173 | -3.67725 | -0.68017 |
| H |  | 2.011841 | -0.43605 | -2.11807 |
| H |  | 1.574894 | -0.75174 | 1.703441 |
| H |  | 1.771903 | -2.49917 | 1.616194 |
| H |  | 0.184074 | -1.38523 | -2.62334 |
| H |  | -0.29247 | -2.98786 | -2.07062 |
| H |  | -1.45125 | -1.66972 | -2.04209 |
| H |  | -0.29079 | -0.00569 | 0.99347 |
| H |  | -0.28009 | -2.46266 | 1.52824 |
| H |  | -0.63203 | -3.5938 | 0.217516 |
| H |  | 1.655834 | 1.7527 | -1.5367 |
| H |  | 3.797997 | -1.35695 | 2.376257 |
| H |  | 4.107404 | -2.27457 | 0.926007 |
| H |  | -0.42918 | 0.673204 | -1.97044 |
| H |  | -0.5931 | 1.842261 | -0.66173 |
| H |  | -2.55355 | -2.13756 | 1.57273 |
| H |  | 5.471084 | -0.76869 | -1.93882 |
| H |  | 4.60663 | -2.05831 | -1.15368 |
| H |  | 3.952507 | -1.32563 | -2.61568 |
| H |  | 3.599434 | 0.691619 | 1.164336 |
| H |  | 3.922891 | 1.346621 | -2.49882 |
| H |  | 3.551834 | 2.126078 | -0.98038 |
| H |  | 2.415661 | 1.957079 | 0.578322 |
| H |  | 6.171887 | -0.59618 | 0.259784 |
| H |  | 6.273044 | 1.018127 | -1.74624 |
| H |  | 5.79812 | 2.679387 | -1.48536 |
| H |  | -2.52755 | 1.747446 | -1.31308 |
| H |  | -4.21893 | 1.815465 | -0.89058 |
| H |  | 5.377541 | 0.594969 | 2.956938 |
| H |  | 7.047907 | 0.358117 | 2.435822 |
| H |  | 5.989664 | -1.0344 | 2.649428 |
| H |  | 7.031965 | 1.672382 | 0.438164 |
| H |  | -3.666 | 2.362729 | 1.454712 |
| H |  | -1.95418 | 2.258018 | 1.094866 |
| H |  | -6.29093 | -0.59037 | -0.39439 |
| H |  | -3.99322 | -3.59438 | 2.149399 |
| H |  | -4.08448 | -5.27035 | 1.568828 |
| H |  | -2.50736 | -4.45964 | 1.744425 |
| H |  | 5.477243 | 3.353013 | 0.765226 |
| H |  | -1.9365 | 4.340985 | -0.09993 |
| H |  | -2.77792 | 6.430939 | -0.59632 |
| H |  | -4.44109 | 6.364664 | -1.20816 |
| H |  | -4.14138 | 6.900944 | 0.435103 |
| H |  | -5.42923 | 3.362306 | 0.881984 |
| H |  | -5.73135 | 5.052374 | 1.31674 |
| H |  | -6.01401 | 4.511011 | -0.32684 |

### 2. Figure and Tables

**
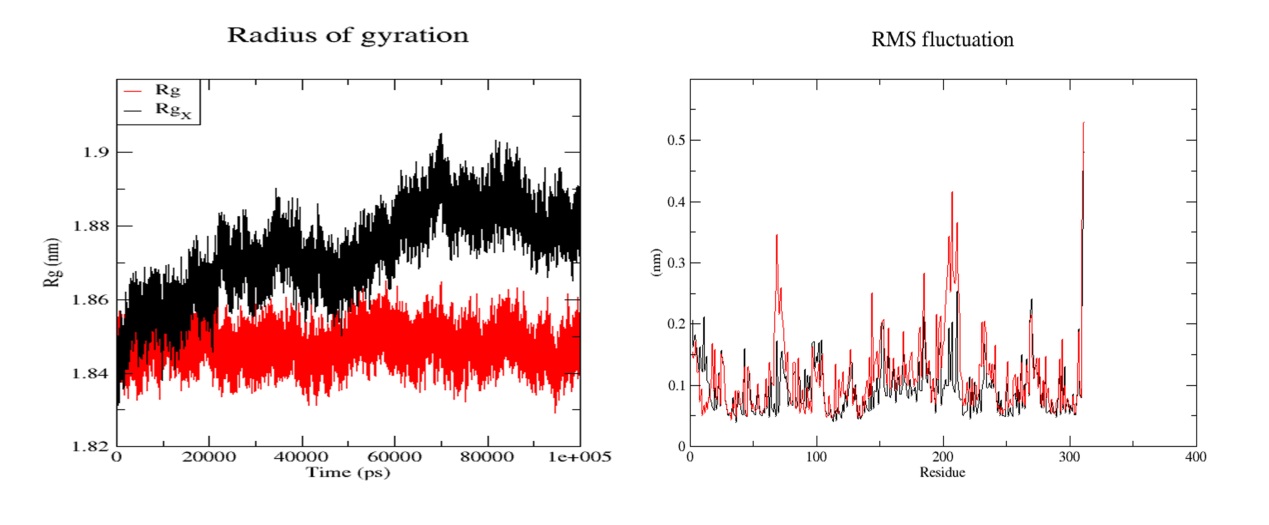
**

**Fig. S1.** The specific information of gyration radius and RMSF plot, red line represented andrographolide-6KHW and black line represented fusid acid-6KHW.

**Table S1** The calculated HOMO(-1) and LUMO(+1) values of title compound.

**AGP**

| **_solvent_** | **HOMO**  **(eV)** | **LUMO**  **(eV)** | **△*E*_HOMO-LUMO_**  **(eV)** | **△*E*_HOMO-LUMO_**  **(kJ/mol)** |
| --- | --- | --- | --- | --- |
| GAS | -8.2532 | -0.4832 | 7.7699 | 749.6893 |
| DMSO | -8.0747 | -0.3083 | 7.7665 | 749.3487 |

**FA**

| **_solvent_** | **HOMO**  **(eV)** | **LUMO**  **(eV)** | **△*E*_HOMO-LUMO_**  **(eV)** | **△*E*_HOMO-LUMO_**  **(kJ/mol)** |
| --- | --- | --- | --- | --- |
| GAS | -7.7898 | -0.3959 | 7.3938 | 713.4024 |
| DMSO | -7.7472 | -0.0124 | 7.7348 | 746.2974 |

**Table S2** Changes in bond angles and distances between the reaction complex (RC) and the transition state (TS).

|  | **Solvent** | **Overall variance (kcal/mol)^2^** | **Positive variance (kcal/mol)^2^** | **Negative variance (kcal/mol)^2^** |
| --- | --- | --- | --- | --- |
| AGP | GAS | 258.33 | 99.48 | 158.85 |
|  | DMSO | 383.98 | 149.99 | 233.98 |
| FA | GAS | 222.60 | 50.60 | 171.99 |
|  | DMSO | 275.79 | 62.49 | 213.30 |

|  | **Solvent** | **Overall average value (kcal/mol)** | **Positive average value (kcal/mol)^2^** | **Negative average value (kcal/mol)** |
| --- | --- | --- | --- | --- |
| AGP | GAS | 2.47 | 12.94 | -16.20 |
|  | DMSO | 2.95 | 17.51 | -21.11 |
| FA | GAS | 1.31 | 8.40 | -12.97 |
|  | DMSO | 1.19 | 10.81 | -16.42 |
